# Adiponectin regulates the circadian rhythm of glucose and lipid metabolism

**DOI:** 10.1530/JOE-22-0006

**Published:** 2022-06-06

**Authors:** Taira Wada, Yukiko Yamamoto, Yukiko Takasugi, Hirotake Ishii, Taketo Uchiyama, Kaori Saitoh, Masahiro Suzuki, Makoto Uchiyama, Hikari Yoshitane, Yoshitaka Fukada, Shigeki Shimba

**Affiliations:** 1 Laboratory of Health Science, School of Pharmacy, Nihon University, Funabshi, Chiba, Japan; 2 Laboratory of Organic Chemistry, School of Pharmacy, Nihon University, Funabshi, Chiba, Japan; 3 Department of Psychiatry, Nihon University School of Medicine, Itabashi-ku, Tokyo, Japan; 4 Tokyo Adachi Hospital, Adachi, Tokyo, Japan; 5 Department of Biological Sciences, School of Science, The University of Tokyo, Bunkyo-ku, Tokyo, Japan

**Keywords:** adiponectin, circadian rhythm, liver, metabolism, VLDL, insulin

## Abstract

Adiponectin is a cytokine secreted from adipocytes and regulates metabolism. Although serum adiponectin levels show diurnal variations, it is not clear if the effects of adiponectin are time-dependent. Therefore, this study conducted locomotor activity analyses and various metabolic studies using the adiponectin knockout (APN (−/−)) and the APN (+/+) mice to understand whether adiponectin regulates the circadian rhythm of glucose and lipid metabolism. We observed that the *adiponectin* gene deficiency does not affect the rhythmicity of core circadian clock genes expression in several peripheral tissues. In contrast, the *adiponectin* gene deficiency alters the circadian rhythms of liver and serum lipid levels and results in the loss of the time dependency of very-low-density lipoprotein-triglyceride secretion from the liver. In addition, the whole-body glucose tolerance of the APN (−/−) mice was normal at CT10 but reduced at CT22, compared to the APN (+/+) mice. The decreased glucose tolerance at CT22 was associated with insulin hyposecretion *in vivo*. In contrast, the gluconeogenesis activity was higher in the APN (−/−) mice than in the APN (+/+) mice throughout the day. These results indicate that adiponectin regulates part of the circadian rhythm of metabolism in the liver.

## Introduction

Adiponectin is a humoral factor that is abundantly secreted by adipocytes (Scherer *et al.* 1995, Hu *et al.* 1996, Maeda *et al.* 1996, Nakano *et al.* 1996). Adiponectin levels are reduced in obese individuals (Arita *et al.* 1999). Because adiponectin is a crucial factor in the regulation of glucose and lipid metabolism, inflammation, and oxidative stress, reduced adiponectin levels play a causal role in the development of insulin resistance, metabolic syndrome, type 2 diabetes, atherosclerosis, and cardiovascular disease (Hotta *et al.* 2000, Lindsay *et al.* 2002, Kumada *et al.* 2003, Pischon *et al.* 2004, Ryo *et al.* 2004, Ohashi *et al.* 2010, Ai *et al.* 2011, Juonala *et al.* 2011). Several studies have shown that the circulating adiponectin levels and the expression level of adiponectin receptors exhibit diurnal variations (Supplementary Fig. 1, see section on supplementary materials given at the end of this article,Gavrila *et al.* 2003, Calvani *et al.* 2004, Yildiz *et al.* 2004, Blüher *et al.* 2005, Gómez-Abellán *et al.* 2010, Tsang *et al.* 2020). However, it is not clear if the effects of adiponectin are time-dependent.

The diurnal rhythms of behavior and physiology are generated by the circadian clock system. In mammals, the central pacemaker of the circadian clock system is located in the suprachiasmatic nucleus (SCN) of the hypothalamus, which responds to the light–dark cycle and regulates the circadian rhythm of behavior (Moore & Eichler 1972, Stephan & Zucker 1972, Ralph *et al.* 1990). Peripheral tissues also have a circadian clock system, which can be driven autonomously (Balsalobre *et al.* 1998, Yamazaki *et al.* 2000, Yoo *et al.* 2004). The SCN clock and the peripheral clocks are synchronized by neural and hormonal stimuli (Kaneko *et al.* 1980, Ueyama *et al.* 1999, Oster *et al.* 2006, Kalsbeek *et al.* 2010). The activity of the peripheral clocks is also influenced by the nutrients in the diet (Iwanaga *et al.* 2005, Eckel-Mahan *et al.* 2013). Several studies have shown a close relationship between the circadian clock system and nutrient metabolism; thus, it is widely established that circadian clock disruptions result in metabolic disorders and ultimately obesity and the related diseases (Rudic *et al.* 2004, Broberger 2005, Turek *et al.* 2005, Buijs & Kreier 2006, Oishi *et al.* 2006, Kennaway *et al.* 2007, Lamia *et al.* 2008, Yang *et al.* 2009, Shimba *et al.* 2011). Conversely, obesity simultaneously decreases the expression of clock genes in each tissue; for example, a high-fat diet can alter the rhythmic expression of clock genes in the liver and the adipose tissue and affect behavioral rhythms (Kohsaka *et al.* 2007).

The levels of several hormones and cytokines show a circadian rhythm (Andrews & Folk 1964, Barter *et al.* 1971, Vaughan *et al.* 1976, Kaneko *et al.* 1980, Arvidson *et al.* 1994, Sinha *et al.* 1996, Bodosi *et al.* 2004, Yu *et al.* 2011). Hormonal rhythms are not merely an output of the central clock, but they also serve as feedback signals to regulate the circadian system. For example, glucocorticoids, secreted from the adrenal gland, directly affect the circadian clock gene expression in several peripheral tissues (Gómez-Abellán *et al.* 2012, Pezük *et al.* 2012). Adrenalectomies shorten re-entrainment in the SCN, lungs, and kidneys resulting in phase shifts (Pezük *et al.* 2012). In addition, several metabolic hormones such as insulin, leptin, and oxyntomodulin are known to be effectors of food entrainment by peripheral tissue clocks (Prosser & Bergeron 2003, Mendoza *et al.* 2011, Tahara *et al.* 2011, Yamajuku *et al.* 2012, Landgraf *et al.* 2015). The detailed relationship between adiponectin and the circadian clock has not been fully elucidated. However, both are deeply involved in the regulation of metabolic activity, and their functions are impaired by obesity. The actions of adiponectin are, at least partly, mediated by AMP-activated protein kinase (AMPK) (Yamauchi *et al.* 2002). AMPK is also involved in the regulation of circadian rhythms (Um *et al.* 2011). These results prompted us to investigate whether adiponectin and the circadian clock are linked and cooperatively involved in metabolic regulation.

## Materials and methods

### Animals

All mice used in the experiments were male and 16–18 weeks old. The *adiponectin* knockout mice (APN (−/−) mice) were purchased from the Jackson Laboratory (Bar Harbor, Maine, USA) (Ma *et al.* 2002). The mice were bread with C57BL/6J mice more than ten times and maintained as the APN (+/−) mice. The APN (−/−) mice were then generated by breeding the APN (+/−) mice. In all experiments, the littermate APN (+/+) mice were used as the control mice. All the mice were maintained under a 12 h light:12 h darkness cycle at 23 ± 1°C with 50 ± 10% relative humidity. Food and water were provided *ad-libitum*. For the experiments under constant darkness (DD), mice were entrained to DD condition for 3 days. 8:00 h, which is zeitgeber time 0, was designated as circadian time (CT) 0. The experimental methods and design adhered to ARRIVE guidelines. The experimental protocol was approved by the Ethics Review Committee for Animal Experimentation of Nihon University (approval nos. AP15P022 (2/3/2016)) and carried out according to relevant guidelines and regulations.

### Locomotor activity

The free-moving activity of the mice was recorded using infrared motion sensors positioned directly above each cage, and the data were continuously recorded using an online system (Melquest Ltd., Toyama, Japan).

### Biochemical analysis of tissue and blood

To measure the liver triglyceride (TG) levels of the mice, lipids were extracted from the tissues using the method described by Carlson (1963). To determine liver cholesterol contents, lipids were extracted using the method described byFolch *et al.* (1957). The levels of TG, total cholesterol, HDL cholesterol, LDL cholesterol, non-esterified fatty acids (NEFA) (FUJIFILM Wako Pure Chemical Co. ltd., Osaka, Japan), insulin (Morinaga Institute of Biological Science, Inc., Kanagawa, Japan), and adiponectin (Otsuka Pharmaceutical Co., Ltd., Tokyo, Japan) were determined using commercial assay kits according to the manufacturer’s instructions.

### Metabolic studies

The glucose tolerance testing (GTT) and the insulin tolerance tests (ITT) were performed by administrating an intraperitoneal injection of dextrose solution (1 g/kg body weight) or insulin (0.5 U/kg body weight; Eli Lilly), respectively. The mice were subjected to fasting for 16 h for the GTT and 6 h for the ITT. Glucose levels were monitored before and after the injection with blood glucose strips (Arkray, Kyoto, Japan). To perform the pyruvate tolerance test (PTT), mice were fasted for 20 h. After intraperitoneal injection of pyruvate (1 g/kg), blood glucose levels were monitored as described above. To measure the very-low-density lipoprotein (VLDL)-TG secretion activity, mice that had fasted for 10 h were treated with tyloxapol (0.5 g/kg body weight). Serum samples were collected at the indicated times, and TG levels were determined as described above.

### Gene expression (quantitative reverse transcription PCR)

Total RNA was extracted using RNAiso Plus (Takara Co., Ltd., Otsu, Japan) according to the manufacturer’s instructions. cDNA was synthesized from 1.0 µg of total RNA using a reverse transcriptase (Toyobo Co., Ltd., Osaka, Japan), and aliquots of cDNA were amplified on a Stratagene MX3000 real-time PCR System using SYBR Green PCR reagents (Promega). Relative gene expression was quantified using the DD threshold cycle (Ct) method. The mRNA expression levels were normalized against 36B4 expression. The primer sequences used are shown in Table 1.

**Table 1:** Primer sequences.

Gene	Forward (5’–3’)	Reverse (5’–3’)
36B4	AAGCGCGTCCTGGCATTGTCT	CCGCAGGGGCAGCAGTGGT
Abca1	TCCTCATCCTCGTCATTCAAACCT	GGACTTGGTAGGACGGAA
Adipo R1	CTTGACGATGCTGAGACCAA	GCTGTGGGGAGCAGTAGAAG
Adipo R2	ACCCACAACCTTGCTTCATC	TAGCCAGCCTATCTGCCCTA
ApoB100	CTGGTTACTGAGCTGAGAGGC	GCTGTCCACACTGAACCAAG
Bmal1	AAGCTTCTGCACAATCCACAGCAC	TGTCTGGCTCATTGTCTTCGTCCA
Clock	TGCCAGCTCATGAAAAGATG	CGCTGCTCTAGCTGGTCTTT
Cry1	CTCGGGTGAGGAGGTTTTCTT	GACTTCCTCTACCGAGAGCTTCAA
Cry2	CTCGTCTGTGGGCATCAAC	ATGGCTGCATCCCGTTCTTT
Dbp	ACCGTGGSGGTGCTAATGAC	TTGTACCTCCGGCTCCAGTA
Fas	TGCTCCCAGCTGCAGGC	GCCCGGTAGCTCTGGGTGTA
Hmg-coar	GATTCTGGCAGTCAGTGGGAA	GTTGTAGCCGCCTATGCTCC
Insig2a	CCCTCAATGAATGTACTGAAGGATT	TGTGAAGTGAAGCAGACCAATGT
Ldlr	GAAGTCGACACTGTACTGACCACC	CTCCTCATTCCCTCTGCCAGCCAT
Mtp	TCAGCGGCTATACAAGCTCAC	CTGGAAGATGCTCTTCTCGC
Per1	AACAGCAGCCACGGTTCTCA	GGTCATCAGAGTGGCCAGGA
Per2	AGAGTGTGGTGTCCCATC	ATGTGCACTAAGGGAGAAGG
Pparα	ATGCCACTACTGCCGTTTTC	GGCCTTGACCTTGTTCATGT
Rev-erbα	CTTCCGTGACCTTTCTCAGC	CAGCTCCTCCTCGGTAAGTG
Rev-erbβ	GCTTCAGGTGGATGGCAGA	TGGCACAAACATGCCAACA
Srebp-1c	GGAGCCATGGATTGCACATT	GCTTCCAGAGAGGAGGCCAG
Srebp-2	CACCTGTGGAGCAGTCTCAA	TGGTAGGTCTCACCCAGGAG
T-cadherin	CATCGAAGCTCAAGATATGG	GATTTCCATTGATGATGGTG

### Statistical analysis

When applicable, the results are presented as means ± s.d. The statistical analyses were performed using a Student’s *t*-test or a two-way ANOVA with Tukey’s* post hoc* test. Circadian rhythmicity was assessed using CircWave software (Oster *et al.* 2006). Statistically significance was set at *P* < 0.05.

## Results

### Locomotor activity and food intake

**Figure 1:**
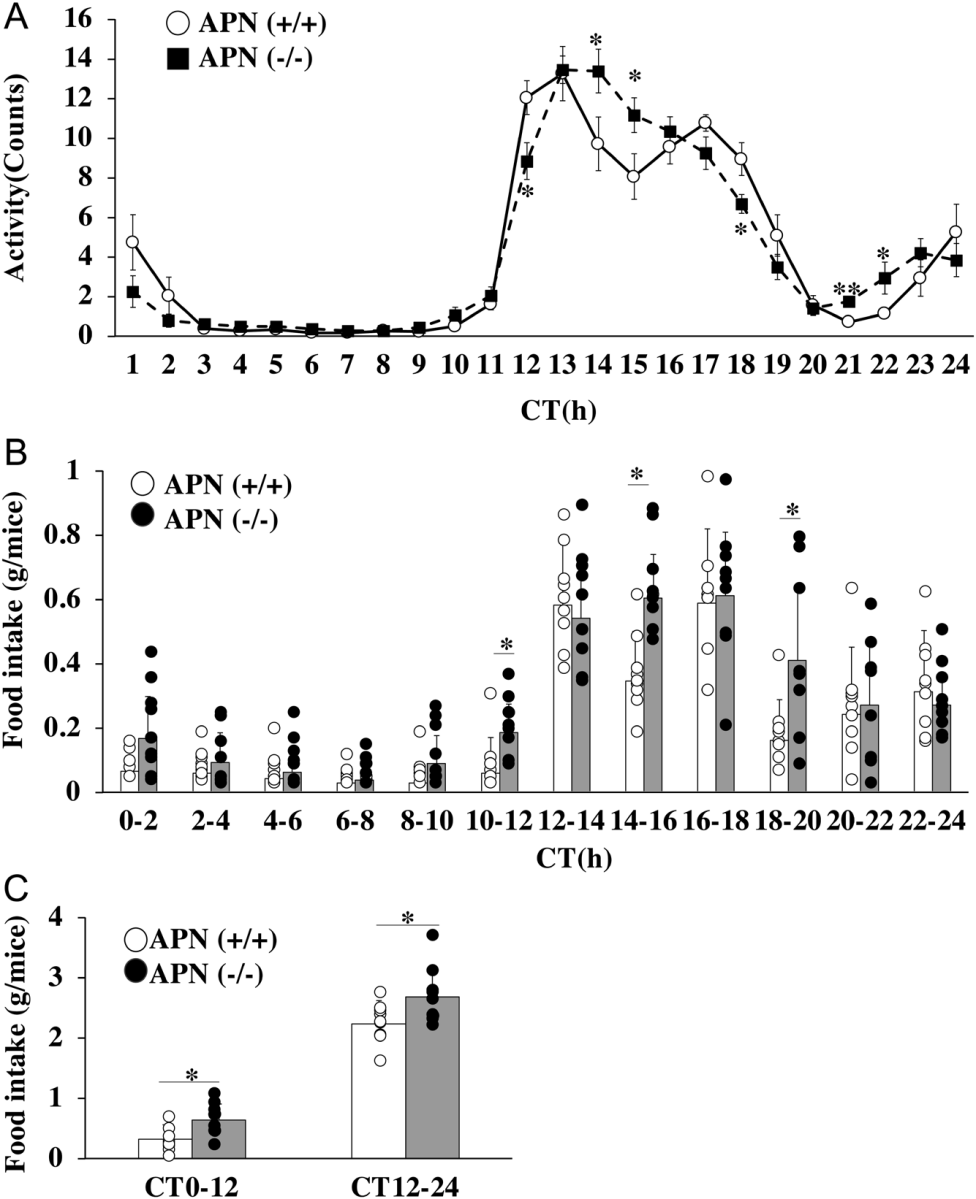
Deletion of the *adiponectin* gene alters the profiles of daily free-moving activity and food intake. APN (+/+) mice and APN (−/−) mice were individually housed in constant darkness (DD). (A) Locomotor activity. (B) Daily food intake profiles. (C) Average of food intake for 12-h intervals. The data are represented as the means ± s.d. (*n* = 9). *P < 0.05, **P < 0.01 relative to the APN (+/+) mice at the same timepoint.

In the first set of experiments, the locomotor activity and the food intake of the APN (−/−) mice were compared to those of the APN (+/+) mice. As shown in Fig. 1A, both the APN (+/+) mice and the APN (−/−) mice exhibited maximal locomotor activity between CT12 and CT20. However, the pattern of the activity was slightly different between the two genotypes. The activity pattern of the APN (+/+) mice was a bimodal between CT12 and CT17, whereas the APN (−/−) mice showed a unimodal locomotor activity pattern in that period (Fig. 1A). The differences in the feeding pattern between the APN (+/+) mice and the APN (−/−) mice are similar to this behavioral pattern, that is, the amount of food intake by the APN (+/+) mice decreased between CT14 and CT16, showing a bimodal pattern, whereas the APN (−/−) mice were still feeding at that time period (Fig. 1B). Thus, the total food intake of the APN (−/−) mice was more than that of the APN (+/+) mice (Fig. 1C).

### Expression of clock genes in the peripheral tissues

**Figure 2:**
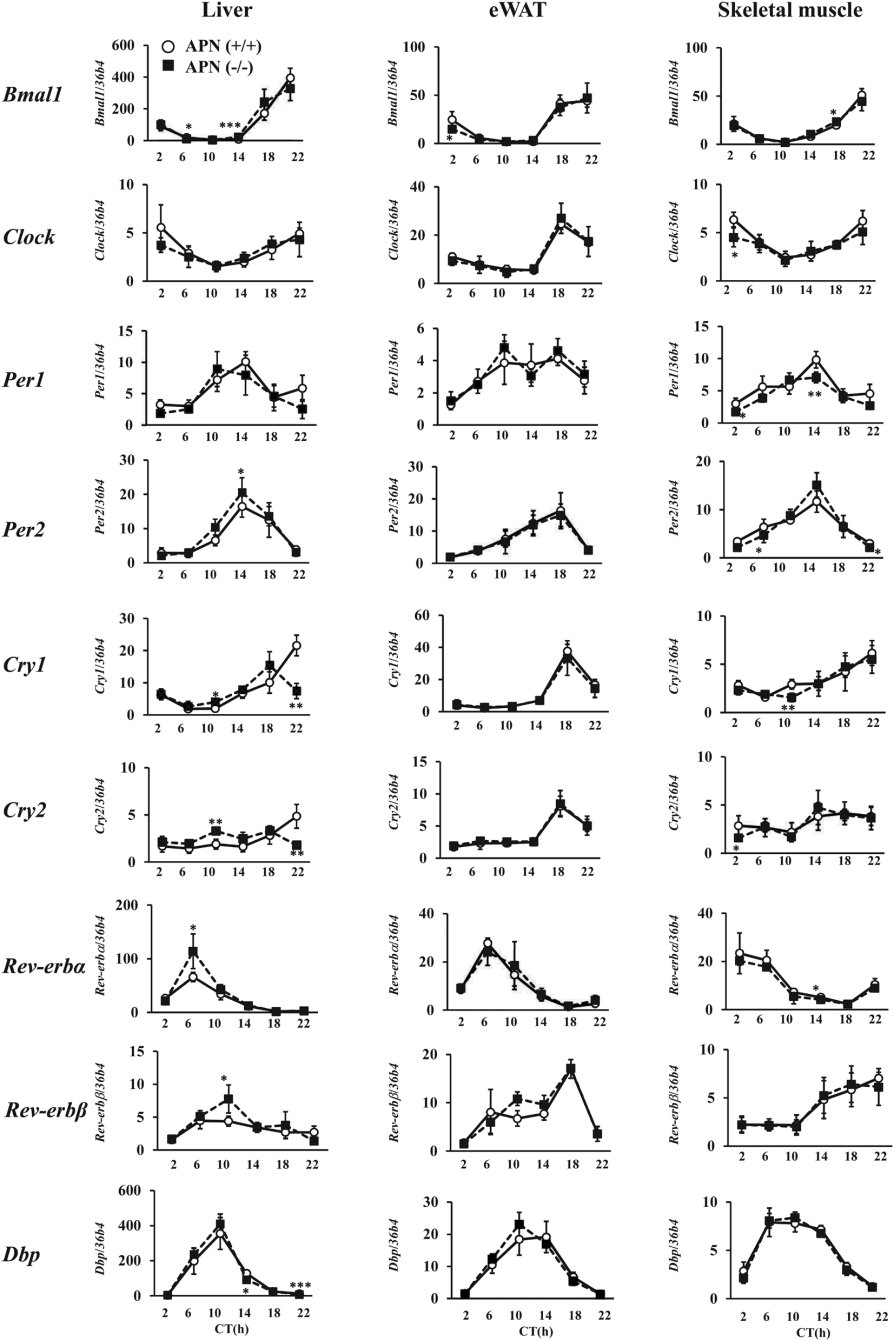
Deletion of the *adiponectin* gene has no effects on the expression of the clock genes in the peripheral tissues. Gene expression in the tissues of the mice housed under DD conditions was analyzed by quantitative reverse transcription PCR. The data are represented as the means ± s.d. (*n* = 5). *P < 0.05, **P < 0.01, ***P < 0.001 relative to the APN (+/+) mice at the same timepoint.

The expression patterns of clock genes and clock-related genes in the liver, the epididymal adipose tissue (eWAT), and the skeletal (gastrocnemius) muscle of the APN (+/+) mice and the APN (−/−) mice were determined. The results in Fig. 2 showed that the *adiponectin* gene deficiency seems largely unaffected on the level and the rhythmicity of the clock genes expression in the eWAT and the skeletal muscle (Fig. 2). In contrast, a part of the hepatic clock genes expression showed the changes with the *adiponectin* deficiency. The expression peaks of *Per2*, *Rev-erb α*, and *Rev-erb β* in the APN (−/−) mice were higher than those in the APN (+/+) mice (Fig. 2). Also, the expression peaks of *Cry1* and *Cry2* at CT22 observed in the APN (+/+) mice were not seen in the APN (−/−) mice (Fig. 2).

### Lipid metabolism

**Figure 3:**
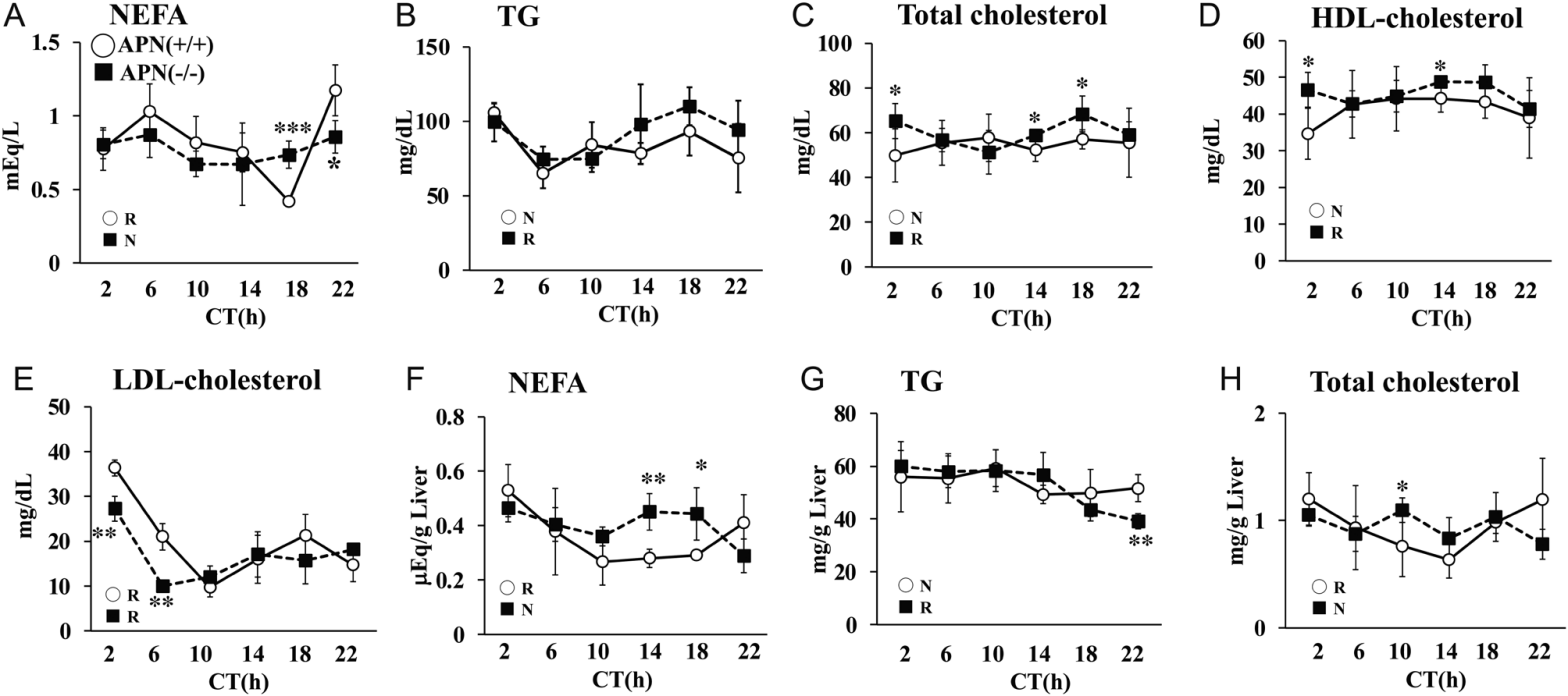
Deletion of the adiponectin gene alters daily profiles of lipid level in the liver and serum. Serum samples (A, B, C, D, and E) and liver samples (F, G, and H) were prepared from male mice housed under DD conditions. (A) NEFA. (B) TG. (C) Total cholesterol (blood). (D) HDL-cholesterol. (E) LDL-cholesterol. (F) NEFA. (G) TG. (H) Total cholesterol (liver). The data are represented as the means ± s.d. (*n* = 8). *P < 0.05, **P < 0.01, ***P < 0.001 relative to the APN (+/+) mice at the same timepoint. The rhythmicity of lipid level was calculated using Circwave software. R, rhythmic expression (*P* < 0.05); N, non-rhythmic expression (*P* > 0.05).

Next, we investigated the circulating lipid level in the mice (Fig. 3A, B, C, D and E). Circadian variation in the serum NEFA level was observed in the APN (+/+) mice but not in the APN (−/−) mice (Fig. 3A). In contrast, the levels of TG, total cholesterol, and HDL-cholesterol remained constant throughout the day in the APN (+/+) mice but showed circadian variations in the APN (−/−) mice (Fig. 3B, C and D). The LDL-cholesterol levels showed circadian variations in both the APN (+/+) mice and the APN (−/−) mice, but the amplitude during the 24-h period was reduced in the APN (−/−) mice (Fig. 3E). In addition, the level of circulating LDL-cholesterol in the APN (−/−) mice was significantly lower than that in the APN (+/+) mice between CT2-6 (Fig. 3E).

Figure 3F, G, and H shows the lipid levels in the liver of the APN (+/+) mice and the APN (−/−) mice. The APN (+/+) mice showed marked circadian variations in the levels of NEFA and total cholesterol (Fig. 3F and H). In contrast, the levels were nearly constant throughout the day in the APN (−/−) mice (Fig. 3F and H). The APN (+/+) mice showed constant levels of TG throughout the day (Fig. 3G). In contrast, the APN (−/−) mice showed a gradual decrease in the TG level after CT14, and the level at CT22 was significantly lower than that of the APN (+/+) mice (Fig. 3G).

**Figure 4:**
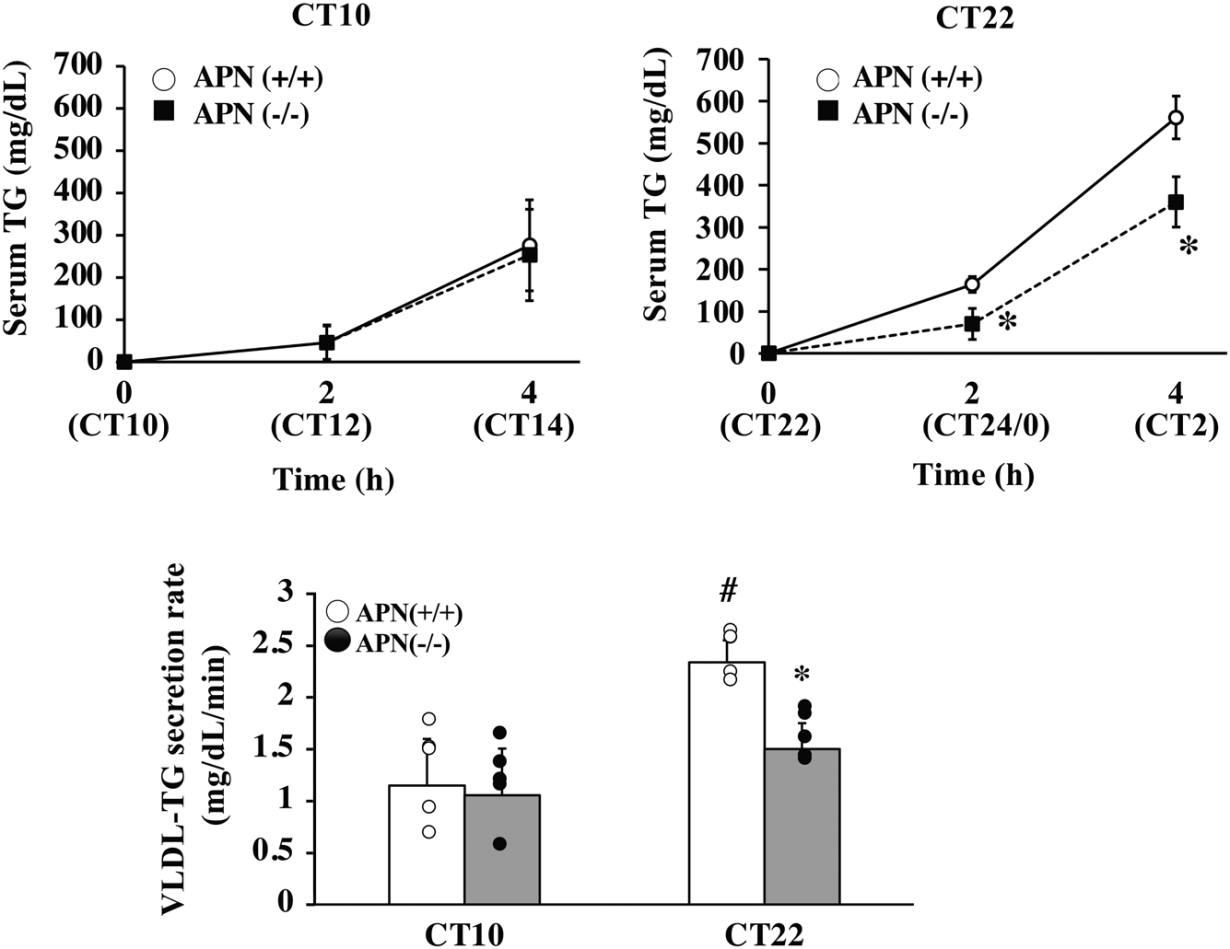
Deletion of the *adiponectin* gene impairs VLDL secretion activity at CT22 but not at CT10. Mice were treated with tyloxapol at CT10 or CT22, and the serum samples were prepared at the indicated time. Top, serum TG level after tyloxapol treatment. Bottom, the area under the curve (AUC) was calculated for each group. The data are represented as the means ± s.d. (*n* = 5). ^#^*P* < 0.05 relative to the APN (+/+) mice at CT10. *P < 0.05 relative to the APN (+/+) mice at the same timepoint.

The VLDL-TG secretion activity in the APN (−/−) mice was comparable to that in the APN (+/+) mice at CT10 (Fig. 4). At CT22, the VLDL-TG secretion activity in the APN (+/+) mice increased, whereas the activity in the APN (−/−) mice was nearly the same as that observed at CT10 (Fig. 4).

### Gene expression related to lipid metabolism

**Figure 5:**
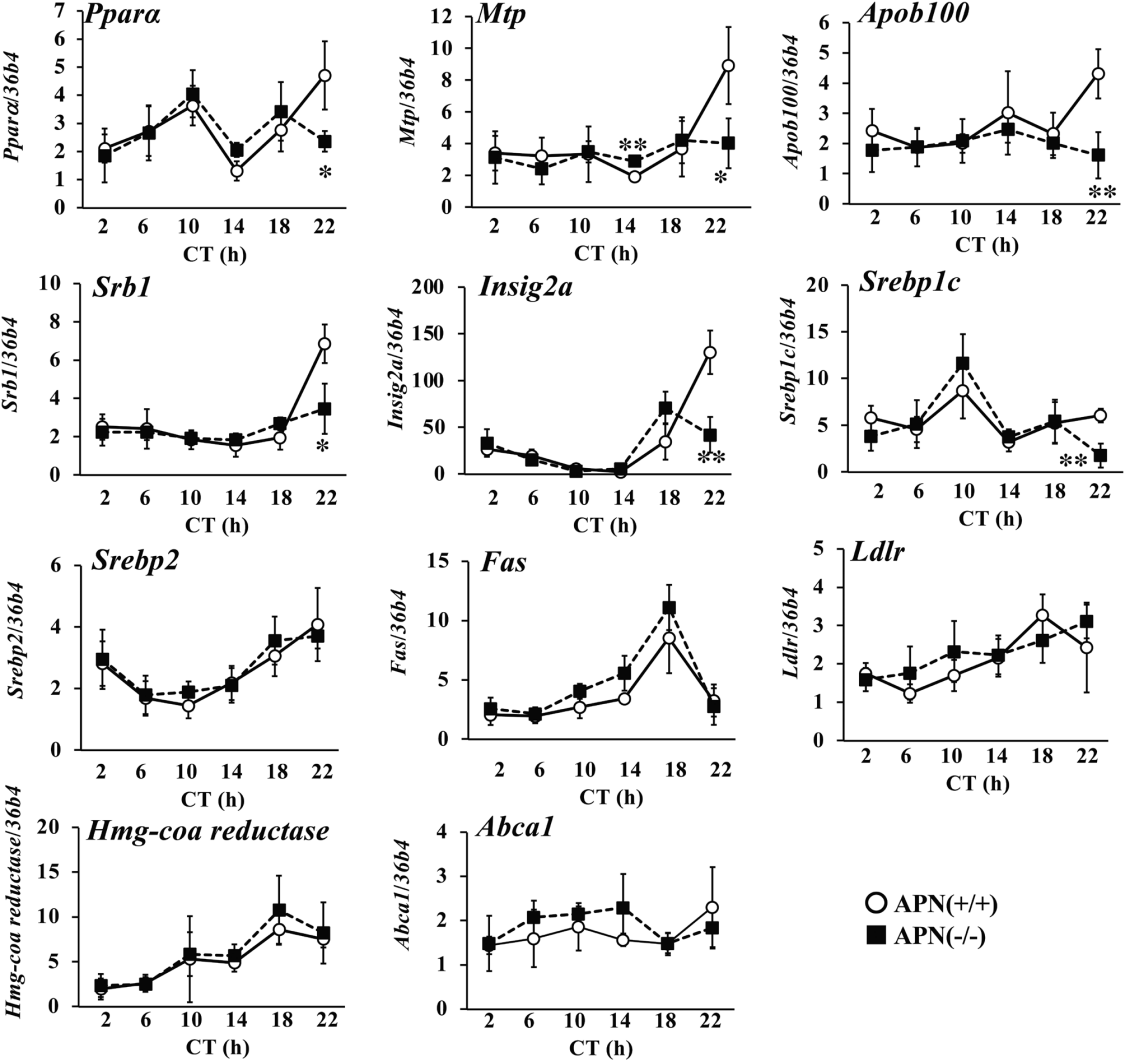
Deletion of the *adiponectin gene* alters the daily profiles of the gene expression related to lipid metabolism in the liver. Gene expression in the livers of the mice housed under DD conditions was analyzed by quantitative reverse transcription PCR. The data are represented as the means ± s.d. (*n* = 5). *P < 0.05, **P < 0.01, relative to the APN (+/+) mice at the same timepoint.

To gain the insight by which deficiency of *adiponectin* gene dysregulates the diurnal variation of lipid metabolism, the expression of genes related to lipid metabolism in the liver was determined. PPARα, which is induced and activated through the adiponectin receptor, mediates the actions of adiponectin in the liver (Yamauchi *et al.* 2003). The expression levels of *Pparα* and its target genes (*Mtp*, *Apob100*, *Srb1*, and *Insig2a*) showed circadian changes and peaked at CT22 in the APN (+/+) mice. However, these expression peaks at CT22 were not observed in the APN (−/−) mice (Fig. 5). The expression level of *Srebp1c* in the APN (−/−) mice was lower than that in the APN (+/+) mice at CT 22 (Fig. 5). There were no differences in the expression levels of the other genes investigated between the two genotypes (Fig. 5).

### Glucose metabolism

**Figure 6:**
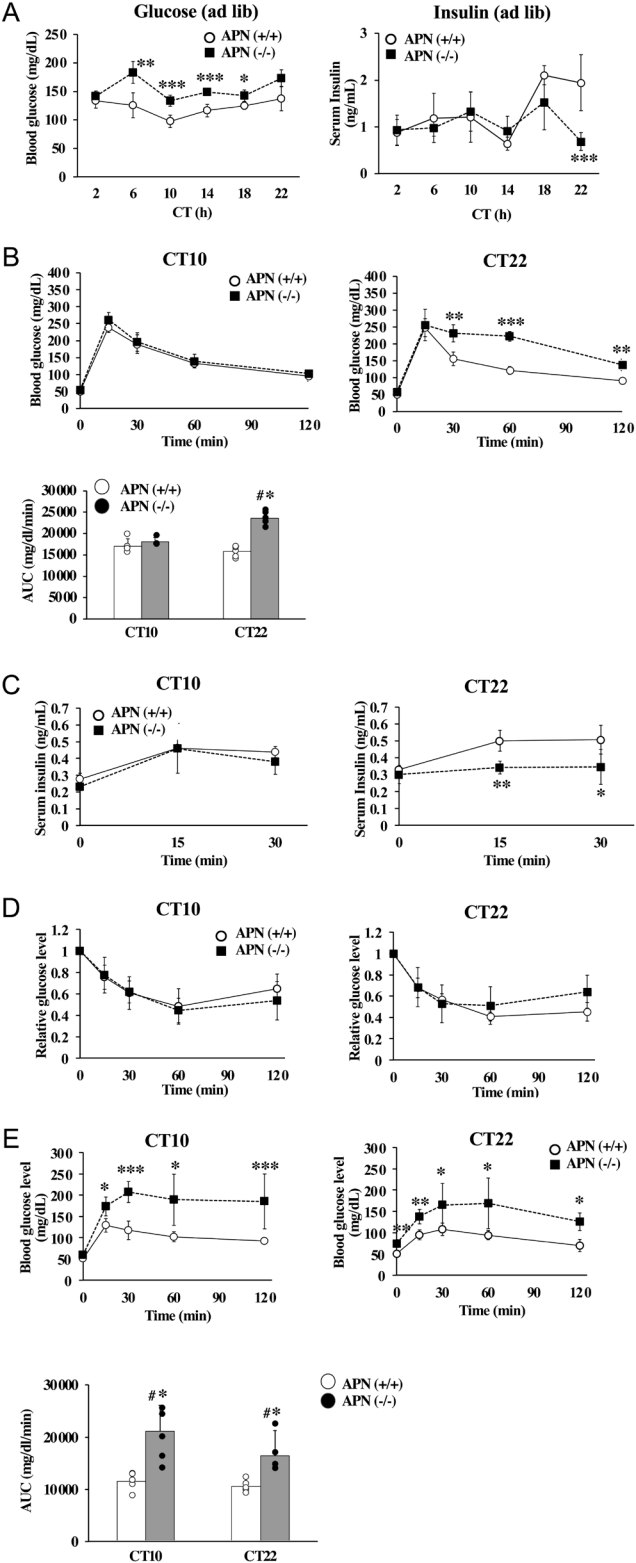
Deletion of the *adiponectin gene* increases blood glucose level in mice. (A) Left, daily blood glucose levels of the mice. Right, daily serum insulin levels of the mice. *P < 0.05, **P < 0.01, ***P < 0.001 relative to the APN (+/+) mice at the same timepoint. (B) Top, glucose tolerance test. **P < 0.01, ***P < 0.001, relative to the APN (+/+) mice at the same timepoint. Bottom, the AUC was calculated for the respective group shown in the top panel. ^#^*P* < 0.05, relative to the APN (+/+) mice at CT10. *P < 0.05, relative to the APN (+/+) mice at the same timepoint. (C) Serum insulin level of the mice during the glucose tolerance test of mice. *P < 0.05, **P < 0.01, relative to the APN (+/+) mice at the same timepoint. (D) Insulin tolerance test. (E) Top, pyruvate tolerance test. *P < 0.05, **P < 0.01, relative to the APN (+/+) mice at the same timepoint. Bottom, the AUC was calculated for the respective group shown in the top panel. The data are represented as the means ± s.d. (*n* = 5). ^#^*P* < 0.05, relative to the APN (+/+) mice at CT10. * *P* < 0.05, relative to the APN (+/+) mice at the same timepoint.

The blood glucose levels of the APN (−/−) mice were higher than those of the APN (+/+) mice throughout the day (Fig. 6A, left), while the significant differences in the blood insulin levels between the two genotypes were observed only at CT22 (Fig. 6A, right). The GTT showed that the whole-body glucose disposal rate of the APN (−/−) mice was comparable to that of the APN (+/+) mice at CT10 but was significantly slower than that of the APN (+/+) mice at CT22 (Fig. 6B). During the GTT, the insulin level in the APN (−/−) mice was similar to that of the APN (+/+) mice at CT10, but it was significantly lower than that of the APN (+/+) mice at CT22 (Fig. 6C). The ITT score for the APN (−/−) mice was similar to that of the APN (+/+) mice, both at CT10 or CT22 (Fig. 6D). The gluconeogenesis activity of both the APN (−/−) mice and the APN (+/+) mice was determined with the PTT at CT10 and CT22. As shown in Fig. 6E, the blood glucose levels in the APN (−/−) mice were higher after pyruvate loading than those of the APN (+/+) mice, regardless of the time of the test.

### Gene expression of adiponectin-binding proteins

**Figure 7:**
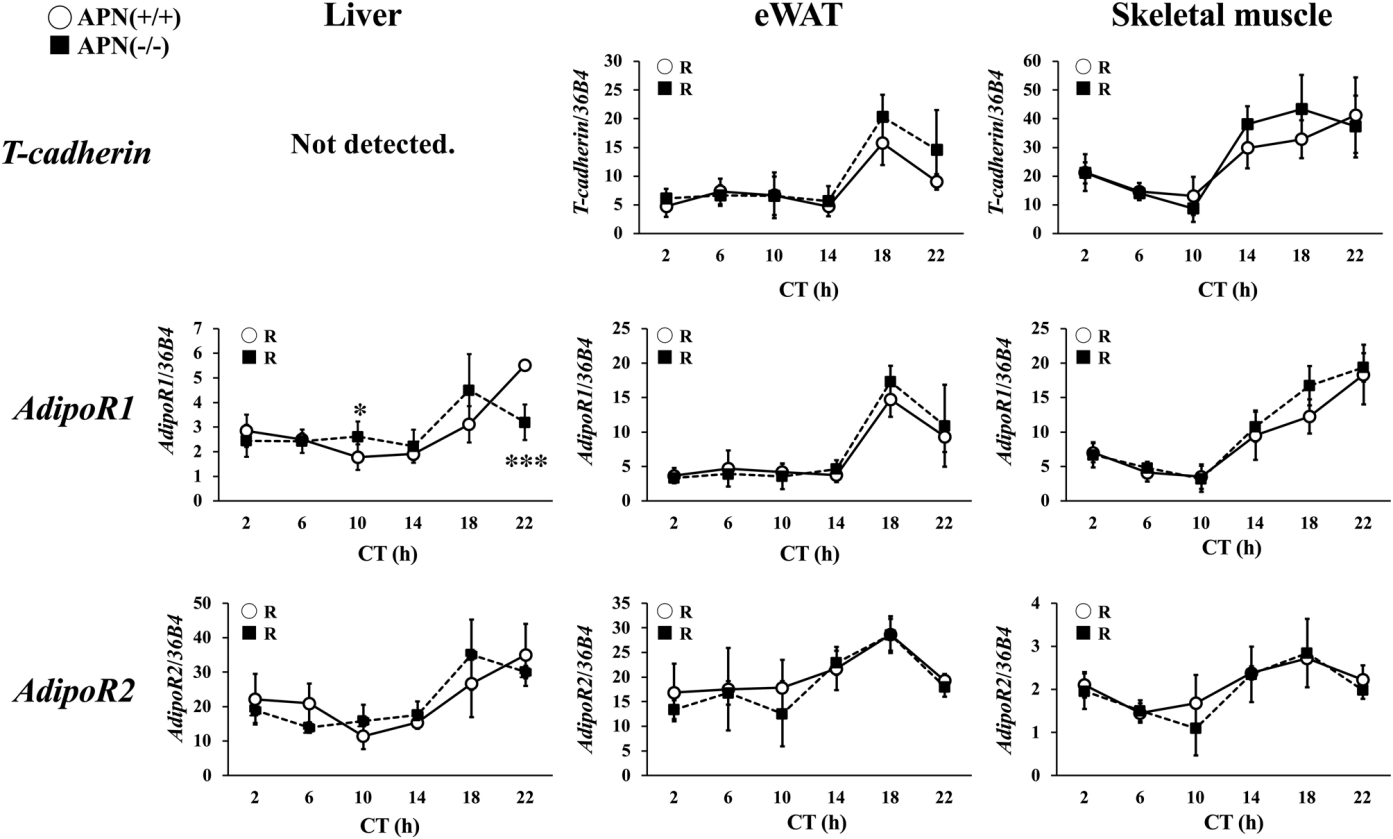
Adiponectin-binding protein gene expression exhibits circadian rhythms in the liver. Gene expression in the liver of the mice housed under DD conditions was analyzed by quantitative reverse transcription PCR. The data are represented as the means ± s.d. (*n* = 5). *P < 0.05, ***P < 0.001, relative to the APN (+/+) mice at the same timepoint. The rhythmicity of each mRNA was calculated using Circwave software. R, rhythmic expression (*P* < 0.05).

Figure 7 shows the expression levels of specific adiponectin binding proteins such as adiponectin receptor (AdipoR)-1 and -2 and T-cadherin. In the eWAT and the skeletal muscle, the expression levels of these adiponectin binding proteins showed circadian variations in both the APN (+/+) mice and the APN (−/−) mice (Fig. 7). However, the *adiponectin* gene deficiency had no effects on the level and the rhythmicity of these adiponectin binding proteins (Fig. 7). In the liver, no *T-cadherin* expression was detected (Fig. 7). The expression level of *AdipoR1* and* 2* in the liver showed circadian variations in both the APN (+/+) mice and the APN (−/−) mice. The expression levels of *AdipoR1* and *AdipoR2* in the liver peaked at CT22 in the APN (+/+) mice and at CT18 in the APN (−/−) mice (Fig. 7).

## Discussion

Adiponectin is a crucial factor in the regulation of glucose and lipid metabolism. The circulating adiponectin levels exhibit a mild diurnal variation (Supplementary Fig. 1,Gavrila *et al.* 2003, Calvani *et al.* 2004, Yildiz *et al.* 2004, Tsang *et al.* 2020). However, it has not been elucidated if the effects of adiponectin are time-dependent. The present study showed that adiponectin regulates the secretion of VLDL-TG and insulin in a time-dependent manner (Figs 4 and 6C). Also, the *adiponectin* gene deficiency altered the circadian rhythm of metabolic parameters in the liver and blood (Fig. 3). Consequently, we are led to conclude that adiponectin exerts time-specific effects on the metabolic regulation in the liver.

The detailed mechanism by which adiponectin exerts its time-specific effects on metabolism is not clear. A series of studies revealed that the binding proteins for adiponectin play crucial roles in adiponectin actions. Three types of binding proteins for adiponectin have been identified: T-cadherin, calreticulin, and AdipoRs. Recent studies strongly suggest that T-cadherin is the actual binding protein for adiponectin in peripheral tissues. However, T-cadherin is not expressed in the liver (Fig. 7) (Kita *et al.* 2019). Calreticulin may acts as an ‘eat me’ signal and the roles in metabolic regulation have not been identified (Gardai *et al.* 2005). Several studies showed that AdipoR1 and R2 are the major binding protein for adiponectin and mediate the adiponectin signaling in the liver (Yamauchi *et al.* 2003, 2007). It has been reported that expression levels of *adipoRs* have daily rhythms (Blüher *et al.* 2005, Gómez-Abellán *et al.* 2010). The results of this study also showed that the expression of *AdipoR1* and *AdipoR2* exhibits a circadian rhythm, with maximum expression at CT22, in the liver (Fig. 7). Also, the expression level of AdipoR2 signaling target genes such as *Ppara* peaked at CT22 in the liver of the APN (+/+) mice, but this increased expression was not observed in the liver of the APN (−/−) mice (Fig. 4). Most of the differences in the metabolic phenotypes between the APN (+/+) mice and the APN (−/−) mice were observed at CT22 in this study (Figs 3, 4, 5, and 6). CT22 is also the time when the serum adiponectin level is at its highest. (Supplementary Fig. 1). Taken together, these results suggest that the coincidence of diurnal variation of serum adiponectin level and the hepatic *AdipoRs* expression level may generate the time-dependent actions of adiponectin in the liver.

This study shows that a deficiency of *adiponectin* altered a part of the expression of clock genes in the liver (Fig. 2). Several studies showed that the clock genes are involved in the regulation of metabolic activity (Kolbe *et al.* 2019, De Assis & Oster 2021, Guan & Lazar 2021). On the other hand, recent studies revealed that the autonomous clock output alone does not appear to contribute to circadian liver homeostasis (Sato *et al.* 2017, Koronowski *et al.* 2019). Also, the rhythmicity of a part of gene expressions in the liver can be generated without being regulated by the core clock genes (Greenwell *et al.* 2019). Thus, the clock genes may participate in the regulation of the time-dependent action of adiponectin in the liver, but the extent to which the clock genes are involved in this regulation is unknown at this stage.

Although this study focused on the roles of adiponectin in the regulation of metabolism in the peripheral tissues, adiponectin also acts on the CNS. As shown in Fig. 1B, the rhythmicity of the feeding of the APN (−/−) mice was altered, that is, the pattern of feeding in the APN(+/+) mice was bimodal between CT13 and CT17, whereas the APN (−/−) mice did not show a transient decrease in that time period and thus increased their food intake (Fig. 1B). This increase in food intake in the APN (−/−) mice is associated with disrupted transcript rhythms of clock and appetite-regulating genes in the mediobasal hypothalamus (Tsang *et al.* 2020). Greenwell *et al.* reported that the daily rhythm of food intake significantly contributes to rhythmic gene expression in the liver without altering core clock gene oscillations (Greenwell *et al.* 2019). Therefore, it could be possible that adiponectin regulates the circadian rhythm of the liver metabolism by acting on the CNS to control food intake.

Although results in this study showed that some of the actions of adiponectin are time-specific, there are some effects of the *adiponectin* gene deficiency that are time-independent. As shown in Fig. 6A, the blood glucose levels in the APN (−/−) mice were higher than those in the APN (+/+) mice throughout the day. Additionally, gluconeogenesis activity as judged by the PTT of the APN (−/−) mice was higher than that of the APN (+/+) mice, regardless of the time of the test (Fig. 6E). Since *adiponectin* deficiency can contribute to hepatic insulin resistance, it should also be considered that the increased gluconeogenesis activity in the APN (−/−) mice could be secondary to the knockout itself. An acute increase in circulating adiponectin levels lowers hepatic glucose production without affecting peripheral glucose uptake, indicating that adiponectin directly regulates gluconeogenesis activity (Berg *et al.* 2001, Combs *et al.* 2001). Therefore, although we do not exclude the possibility that the increase of gluconeogenesis activity throughout a day in APN (−/−) mice is a part of the secondary effects of *adiponectin* deficiency, we would argue adiponectin may regulate blood glucose levels by controlling gluconeogenesis activity in a time-independent manner.

Hashinaga *et al.* reported that KK-Ta mice, which are a polygenic model of metabolic syndrome with hypoadiponectinemia, have a shorter activity period under constant dark conditions and dampened circadian locomotor rhythms with increased light-phase activity relative to controls (Hashinaga *et al.* 2013). Notably, clock gene rhythms are phase-advanced in the liver and the skeletal muscle of these mice. The introduction of the human adiponectin transgene into the livers of KK-Ta mice restores locomotor rhythmicity, as well as the clock gene phase in the liver. Conversely, we observed that the phase length of the locomotor activity of the APN (−/−) mice was largely comparable to that of the APN (+/+) mice (Fig. 1 and Tsang *et al.* 2020). Behavioral and molecular circadian rhythms are disrupted by metabolic disorders (Kohsaka *et al.* 2007). Therefore, the restoration of the locomotor activity and the clock gene expression in KK-Ta mice with the introduction of the adiponectin gene is most likely primarily due to adiponectin-induced improvements in the metabolic status of the mice. In short, their results also suggest that decreased adiponectin levels under pathological conditions trigger the disruption of metabolic rhythmicity in the liver.

In conclusion, adiponectin regulates the circadian rhythm of liver metabolism. Adiponectin has been implicated in several pathophysiological responses. Adiponectin levels are decreased under pathological conditions associated with energy metabolism disorders, such as obesity (Arita *et al.* 1999, Hotta *et al.* 2000, Lindsay *et al.* 2002, Kumada *et al.* 2003, Pischon *et al.* 2004, Ryo *et al.* 2004, Ohashi *et al.* 2010, Ai *et al.* 2011, Juonala *et al.* 2011). Simultaneously, reduced adiponectin levels alter the circadian rhythm of metabolism as shown in this study. Disturbance of circadian rhythm exacerbates metabolic disorders, resulting in a further decrease in adiponectin levels. Consequently, the results of this study provide new insights into the regulation of energy metabolism by adiponectin and an opportunity to introduce new concepts into therapeutic strategies for metabolic diseases.

## Supplementary Material

Supplemental Figure 1. Diurnal variation of serum adiponectin in C57Bl/6J mice. Serum samples were prepared from male C57Bl/6J mice (18 weeks old) housed under DD conditions. The data are represented as the means ± SD (n=8).

## Declaration of interest

The authors declare that there is no conflict of interest that could be perceived as prejudicing the impartiality of the research reported.

## Funding

This work is in part supported by MEXT/JSPS KAKENHI (grant number 17H06096, 17K08291, 17K08392, 18K06725, 19H03175, 20K07020, 20K07956, and 21K06536: S S, T W, T U, M S, H Y, Y F), The Science Research Promotion Fund from The Promotion and Mutual Aid Corporation for Private Schools of Japan http://dx.doi.org/10.13039/501100012359 (S S, T W), ‘Private University Research Branding Project’ from MEXT (S S), A grant for cooperative research in the School of Pharmacy, Nihon University http://dx.doi.org/10.13039/100007683 (S S, T W), Multidisciplinary Research Grant from Nihon University http://dx.doi.org/10.13039/100007683 (S S, T W), Research Grants From Japan Foundation http://dx.doi.org/10.13039/501100012629 of Institute for Neuropsychiatry (K S), A grant to encourage and promote research project in the School of Pharmacy, Nihon University http://dx.doi.org/10.13039/100007683 (T W).

## Author contribution statement

Participated in research design, T W, T U, M S, M U, H Y, Y F, and S S; conducted experiments, T W, Y Y, Y T, H I, K S, H Y, and S S; contributed new reagents or analytic tools, T W, T U, H Y, and Y F; performed data analysis, T W, Y Y, M S, M U, H Y, Y F, and S S; wrote or contributed to the writing of the manuscript, T W, Y Y, and S S.
