# Supplementary material for: Adiponectin regulates the circadian rhythm of glucose and lipid metabolism
Source: J Endocrinol. 2022 Jun 6;254(2):121–33. doi: 10.1530/JOE-22-0006 (PMC9354065; doi:10.1530/JOE-22-0006)
Supplement: Supplemental Figure 1. Diurnal variation of serum adiponectin in C57Bl/6J mice. Serum samples were prepared from male C57Bl/6J mice (18 weeks old) housed under DD conditions. The data are represented as the means ± SD (n=8). [file supplementary_figure_1.pdf]

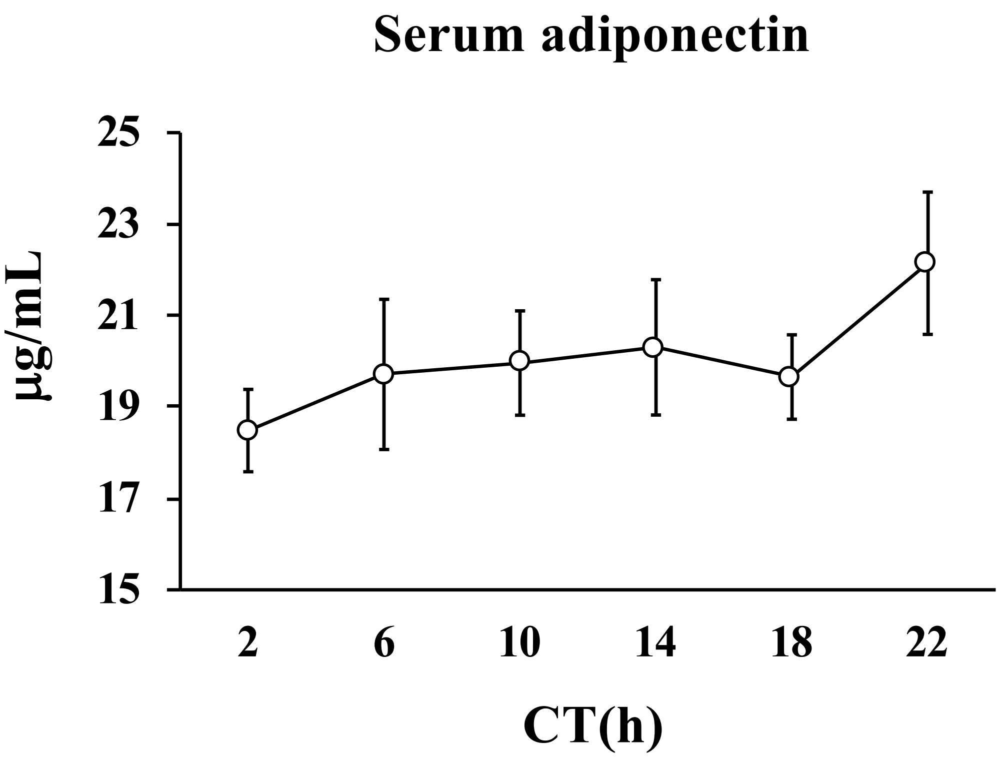

Supplemental Figure 1. Diurnal variation of serum adiponectin in C57Bl/6J mice.
Serum samples were prepared from male C57Bl/6J mice (18 weeks old) housed under DD conditions. The data are represented as the means ± SD (n=8).
